# Pathophysiology of Coagulopathy Induced by Traumatic Brain Injury Is Identical to That of Disseminated Intravascular Coagulation With Hyperfibrinolysis

**DOI:** 10.3389/fmed.2021.767637

**Published:** 2021-11-15

**Authors:** Takeshi Wada, Atsushi Shiraishi, Satoshi Gando, Kazuma Yamakawa, Seitaro Fujishima, Daizoh Saitoh, Shigeki Kushimoto, Hiroshi Ogura, Toshikazu Abe, Toshihiko Mayumi, Junichi Sasaki, Joji Kotani, Naoshi Takeyama, Ryosuke Tsuruta, Kiyotsugu Takuma, Shin-ichiro Shiraishi, Yasukazu Shiino, Taka-aki Nakada, Kohji Okamoto, Yuichiro Sakamoto, Akiyoshi Hagiwara, Satoshi Fujimi, Yutaka Umemura, Yasuhiro Otomo

**Affiliations:** ^1^Division of Acute and Critical Care Medicine, Department of Anesthesiology and Critical Care Medicine, Hokkaido University Faculty of Medicine, Sapporo, Japan; ^2^Emergency and Trauma Center, Kameda Medical Center, Kamogawa, Japan; ^3^Department of Acute and Critical Care Medicine, Sapporo Higashi Tokushukai Hospital, Sapporo, Japan; ^4^Department of Emergency Medicine, Osaka Medical and Pharmaceutical University, Takatsuki, Japan; ^5^Center for General Medicine Education, Keio University School of Medicine, Tokyo, Japan; ^6^Division of Traumatology, Research Institute, National Defense Medical College, Tokorozawa, Japan; ^7^Division of Emergency and Critical Care Medicine, Tohoku University Graduate School of Medicine, Sendai, Japan; ^8^Department of Traumatology and Acute Critical Medicine, Osaka University Graduate School of Medicine, Suita, Japan; ^9^Department of Emergency and Critical Care Medicine, Tsukuba Memorial Hospital, Tsukuba, Japan; ^10^Health Services Research and Development Center, University of Tsukuba, Tsukuba, Japan; ^11^Department of Emergency Medicine, School of Medicine, University of Occupational and Environmental Health, Kitakyushu, Japan; ^12^Department of Emergency and Critical Care Medicine, Keio University School of Medicine, Tokyo, Japan; ^13^Division of Disaster and Emergency Medicine, Department of Surgery Related, Kobe University Graduate School of Medicine, Kobe, Japan; ^14^Advanced Critical Care Center, Aichi Medical University Hospital, Nagakute, Japan; ^15^Advanced Medical Emergency & Critical Care Center, Yamaguchi University Hospital, Ube, Japan; ^16^Emergency & Critical Care Center, Kawasaki Municipal Hospital, Kawasaki, Japan; ^17^Department of Emergency and Critical Care Medicine, Aizu Chuo Hospital, Aizu, Japan; ^18^Department of Acute Medicine, Kawasaki Medical School, Kurashiki, Japan; ^19^Department of Emergency and Critical Care Medicine, Chiba University Graduate School of Medicine, Chiba, Japan; ^20^Department of Surgery, Center for Gastroenterology and Liver Disease, Kitakyushu City Yahata Hospital, Kitakyushu, Japan; ^21^Emergency and Critical Care Medicine, Saga University Hospital, Saga, Japan; ^22^Center Hospital of the National Center for Global Health and Medicine, Tokyo, Japan; ^23^Division of Trauma and Surgical Critical Care, Osaka General Medical Center, Sumiyoshi, Japan; ^24^Trauma and Acute Critical Care Center, Medical Hospital, Tokyo Medical and Dental University, Tokyo, Japan

**Keywords:** disseminated intravascular coagulation, fibrinolysis, shock, thrombin, trauma-induced coagulopathy, traumatic brain injury

## Abstract

**Background:** Traumatic brain injury (TBI)-associated coagulopathy is a widely recognized risk factor for secondary brain damage and contributes to poor clinical outcomes. Various theories, including disseminated intravascular coagulation (DIC), have been proposed regarding its pathomechanisms; no consensus has been reached thus far. This study aimed to elucidate the pathophysiology of TBI-induced coagulopathy by comparing coagulofibrinolytic changes in isolated TBI (iTBI) to those in non-TBI, to determine the associated factors, and identify the clinical significance of DIC diagnosis in patients with iTBI.

**Methods:** This secondary multicenter, prospective study assessed patients with severe trauma. iTBI was defined as Abbreviated Injury Scale (AIS) scores ≥4 in the head and neck, and ≤2 in other body parts. Non-TBI was defined as AIS scores ≥4 in single body parts other than the head and neck, and the absence of AIS scores ≥3 in any other trauma-affected parts. Specific biomarkers for thrombin and plasmin generation, anticoagulation, and fibrinolysis inhibition were measured at the presentation to the emergency department (0 h) and 3 h after arrival.

**Results:** We analyzed 34 iTBI and 40 non-TBI patients. Baseline characteristics, transfusion requirements and in-hospital mortality did not significantly differ between groups. The changes in coagulation/fibrinolysis-related biomarkers were similar. Lactate levels in the iTBI group positively correlated with DIC scores (rho = −0.441, *p* = 0.017), but not with blood pressure (rho = −0.098, *p* = 0.614). Multiple logistic regression analyses revealed that the injury severity score was an independent predictor of DIC development in patients with iTBI (odds ratio = 1.237, *p* = 0.018). Patients with iTBI were further subdivided into two groups: DIC (*n* = 15) and non-DIC (*n* = 19) groups. Marked thrombin and plasmin generation were observed in all patients with iTBI, especially those with DIC. Patients with iTBI and DIC had higher requirements for massive transfusion and emergency surgery, and higher in-hospital mortality than those without DIC. Furthermore, DIC development significantly correlated with poor hospital survival; DIC scores at 0 h were predictive of in-hospital mortality.

**Conclusions:** Coagulofibrinolytic changes in iTBI and non-TBI patients were identical, and consistent with the pathophysiology of DIC. DIC diagnosis in the early phase of TBI is key in predicting the outcomes of severe TBI.

## Introduction

Traumatic brain injury (TBI) is one of the leading causes of death and disability, with more than 10 million people hospitalized for TBI every year (1). Although primary damage to the brain is inevitable, secondary injuries are potentially avoidable and can affect the therapeutic interventions and outcomes of patients with TBI. TBI-associated coagulopathy is widely recognized as a risk factor for secondary brain damage and contributes to poor clinical outcomes. Two meta-analyses reported that the overall prevalence of TBI-associated coagulopathy was 32.7–35.2%, and a strong association was confirmed between the incidence of coagulopathy and poor outcomes (2, 3). TBI-associated coagulopathy contributes to poor outcomes in both hypocoagulability, leading to the expansion of intracranial hemorrhage, and hypercoagulability, leading to secondary cerebral ischemia due to intravascular thrombosis in the injured brain (4–8). Therefore, elucidation of the pathophysiology of TBI-induced coagulopathy may contribute to improving the outcomes of TBI patients, but no consensus has been reached thus far.

Disseminated intravascular coagulation (DIC) is acknowledged as the primary pathophysiological mechanism of trauma-induced coagulopathy (TIC), which is caused by multiple factors, such as anemia, hemodilution, hypothermia, acidosis, hemorrhagic shock, and serious trauma itself (9). Coagulopathy that occurs in patients with TBI is consistent with the definition of DIC proposed by the International Society on Thrombosis and Haemostasis: the activation of coagulation with the loss of localization and damage to the microvasculature (10, 11). Previous studies have clearly demonstrated consumption coagulopathy and disseminated microvascular thrombosis formation in the brain and other organs, which is the pathophysiology of DIC itself (12, 13).

A recent consensus statement from the International Society on Thrombosis and Haemostasis described that TIC is driven by two distinct and synergic insults: hypovolemic shock due to blood loss and extensive tissue disruption (14). However, it is unclear whether this concept can be applied to patients with TBI, since these patients do not suffer substantial blood loss, indicating that they are less likely to develop hemorrhagic shock.

Therefore, this study aimed to elucidate the pathophysiology of TBI-induced coagulopathy by comparing the coagulofibrinolytic changes in isolated TBI (iTBI) with those in non-TBI trauma, and to determine the associated factors, particularly shock-related factors such as blood pressure and serum lactate levels. To gain a deeper insight into the pathomechanisms of TBI-induced coagulopathy, coagulofibrinolytic changes were compared between iTBI patients with and without DIC, and the clinical significance of DIC diagnosis in patients with iTBI was evaluated.

## Materials and Methods

### Study Design, Setting, and Ethical Approval

This descriptive study was performed as a secondary analysis of a multicenter prospective study conducted by the Japanese Association for Acute Medicine (JAAM) Focused Outcomes Research in Emergency Care in Acute Respiratory Distress Syndrome, Sepsis, and Trauma (FORECAST) study group (15). The JAAM FORECAST TRAUMA study recruited participants between April 1, 2016 and January 31, 2018, from 39 emergency departments (EDs) and intensive-care units (ICUs) in tertiary hospitals and was registered at the University Hospital Medical Information Network Clinical Trial Registry (UMIN-CTR ID: UMIN000019588). This study was approved under the condition that written informed consent was obtained from the patient or next of kin by the JAAM and the Ethics Committee of each hospital (JAAM, 2014-01; Hokkaido University Graduate School of Medicine, Head Institute of the FORECAST group, 014-0307) and was performed in accordance with the Declaration of Helsinki.

### Participants

The JAAM FORECAST TRAUMA study enrolled adult trauma patients with severe trauma injury (aged ≥16 years old) with an Injury Severity Score (ISS) of ≥16 who were directly transported from the scene by emergency medical services. Patients with a history of cardiac arrest and resuscitation, who were receiving anticoagulants, who had hemorrhagic diathesis or coagulopathy due to any cause, or who had been transferred from other hospitals were excluded before registration. The size of the study population was dependent on the study period. All patients were followed up until discharge. Twenty-seven healthy volunteers who were not age- or sex-matched were enrolled to obtain the control values of the measured markers.

### Definition and Diagnosis

Injury severity was assessed using the ISS. A DIC diagnosis was made based on the JAAM DIC diagnostic criteria (16) (Supplementary Table 1). The DIC scores were calculated at 0, 3, and 24 h, and the DIC group was defined as patients who met the DIC criteria at least once during the study period. In the present study, the prothrombin time international normalized ratio (PT-INR) was used as a substitute for the prothrombin time ratio for the diagnosis of DIC. Transfusion of packed red blood cells of more than the estimated circulating blood volume (7.5% of body weight) within 24 h after presentation to the ED met the definition of massive transfusion. Shock was defined as a systemic inflammatory response syndrome criteria were used to assess systemic inflammation (17). A systolic blood pressure of <90 mmHg at the scene or at the ED and lactate levels >2 mmol/L at the ED. The Charlson comorbidity index was used to assess comorbidities (18).

iTBI was defined as an Abbreviated Injury Scale (AIS) score of ≥4 in the head and neck and ≤2 in other body parts. To match the injury severity of iTBI, non-TBI was defined as an AIS score of ≥4 in one body part other than the head and neck and the absence of AIS score of ≥3 in any part affected by trauma.

### Data Collection and Measurements

Immediately after arrival at the ED (0 h) and 3 h after admission (3 h), 15 mL of blood was collected in citrate containing tubes at each sampling point. The samples were immediately centrifuged at 4°C in the laboratories of each hospital, and the obtained plasma was stored at −80°C. All plasma samples were measured at the center laboratory of the LSI Medience Corporation (Tokyo, Japan). We measured the following molecular markers: (1) soluble fibrin (marker of direct thrombin generation) (LA, IATRO SFII; LSI Medience), (2) antithrombin (marker of anti-thrombin) (chromogenic assay, HemosIL Antithrombin LQ; Instrumental Laboratory), (3) protein C (marker of anticoagulation) (LPIA, LPIA-ACE PCII; LSI Medience), (4) plasmin antiplasmin complex (marker of plasmin generation) (LPIA, LPIA-ACE PPI II; LSI Medience), (5) plasminogen activator inhibitor-1 (PAI-1) (marker of inhibition of fibrinolysis) (LA, LPIA-tPAI test; LSI Medience), (6) D-dimer (marker of fibrinolysis) (LPIA, LPIA GENESIS D-dimer; LSI Medience), and (7) circulating activated protein C (APC) (marker of inhibition of thrombin) (EIA, EIA Kit For Activated Protein C Cloud-Clone Corp.). In addition to routine laboratory tests and blood gas analysis, measurements of platelet counts, PT-INR, activated partial thromboplastin time (APTT), fibrinogen, fibrin/fibrinogen degradation products (FDPs), and the FDP/D-dimer ratio, a surrogate marker of fibrin(ogen)olysis, were measured at 0, 3, and 24 h after arrival at the ED.

### Statistical Analyses

Measurements are expressed as the median with the 25th−75th interquartile range or as numbers (percentages). Missing values were used without manipulation. Differences in demographics and measured parameters between the two groups were compared using the Mann–Whitney *U*-test for continuous variables and using either the chi-square test or Fisher's exact test for nominal variables when required. Platelet counts and global markers of coagulation and fibrinolysis were also evaluated using the Mann–Whitney *U*-test. Correlations were evaluated using Spearman's rank test when required. Survival probability curves with and without a DIC diagnosis in iTBI were constructed using the Kaplan–Meier method. The receiver operating characteristic (ROC) curve was constructed, and the area under the ROC curve (AUC) was used to assess the ability of DIC scores to predict in-hospital mortality. Differences were considered statistically significant at a two-tailed *p* < 0.05. SPSS software (version 26; IBM Japan, Tokyo, Japan) was used for all statistical analyses and calculations.

## Results

### Baseline Characteristics, Transfusion, and In-Hospital Mortality

In total, 295 consecutive patients were registered during the study period in the FORECAST TRAUMA cohort. Ultimately, 276 patients who met the eligibility criteria were divided into the iTBI (*n* = 34) and non-TBI (*n* = 40) groups (Supplementary Figure 1).

There were no significant differences in age, sex, comorbidities, or ISS between the groups. In addition, the DIC scores and prevalence at 0 and 3 h did not significantly differ between the groups (Table 1). The requirement for massive transfusion, transfusion volumes, and in-hospital mortality did not significantly differ between the two groups (Table 2).

**Table 1:** Demographics and parameters at the scene and admission to the emergency department in patients with isolated traumatic brain injury and non-traumatic brain injury.

	**Non-TBI**	**iTBI**	***p*** **-value**
	***n*** **= 40**	***n*** **= 34**	
Demographics			
Age (years)	67 (55–79)	59 (48–76)	0.633
Male sex, *n* (%)	27 (67.5)	23 (67.6)	0.989
Charlson comorbidity index	0 (0–1)	0 (0–0)	0.711
ISS	20 (17–26)	17(16–25)	0.196
AIS			
Head	0 (0–0)	4 (4–5)	<0.001
Face	0 (0–0)	0 (0–0)	0.814
Neck	0 (0–0)	0 (0–0)	0.357
Thorax	4 (0–4)	0 (0–0)	<0.001
Abdomen	0 (0–2)	0 (0–0)	0.001
Spine			
Cervical	0 (0–0)	0 (0–0)	0.019
Thoracic	0 (0–0)	0 (0–0)	0.019
Lumber	0 (0–0)	0 (0–0)	0.105
Upper extremity	0 (0–0)	0 (0–0)	0.022
Lower extremity	0 (0–2)	0 (0–0)	<0.001
External	0 (0–1)	0 (0–0)	0.048
DIC 0 h, *n* (%)	8 (20.0)	9 (26.5)	0.510
DIC score 0 h	3 (2–3)	3 (1–4)	0.920
DIC 3 h, *n* (%)	10 (28.6)	10 (38.4)	0.416
DIC score 3 h	3(1–4)	3(1–4)	0.875
DIC 24 h, *n* (%)	10 (26.3)	3 (11.1)	0.131
DIC score 24 h	3 (1–4)	3 (0–3)	0.042
SIRS criteria	2 (1–2)	2 (1–3)	0.205
Shock (ABP) at scene, *n* (%)	6 (15.4)	3 (8.8)	0.489
Shock (ABP) at ED, *n* (%)	8 (20.0)	1 (2.9)	0.033
Shock (lac), *n* (%)	21 (56.8)	20 (69.0)	0.310
Tranexamic acid, *n* (%)	8 (20.0)	14 (41.2)	0.047
At the scene			
Systolic blood pressure (mmHg)	119 (103–136)	150 (121–169)	0.004
Diastolic blood pressure (mmHg)	72 (63–87)	80 (65–104)	0.187
Heart rate (bpm)	83 (69–99)	84 (74–97)	0.747
Respiratory rate (breaths/min)	20 (18–24)	18 (18–20)	0.001
At the ED			
Systolic blood pressure (mmHg)	140 (107–151)	144 (131–161)	0.007
Diastolic blood pressure (mmHg)	77 (6,693)	84 (71–95)	0.110
Heart rate (bpm)	82 (65–93)	84 (72–97)	0.520
Respiratory rate (breaths/min)	20 (18–26)	18 (16–20)	<0.001
Lactate (mmol/L)	1.7 (1.1–3.1)	3.1 (1.7–4.3)	0.372
Body temperature (°C)	36.4 (36.1–36.8)	36.3 (35.9–37.0)	0.853

*Reported proportions (counts) for categorical variables and medians (interquartile ranges) for continuous variables. Shock (ABP) represents a systolic blood pressure of <90 mmHg. Shock (lac) represents lactate levels of >2 mmol/L in the emergency department (ED)*.

*AIS, Abbreviated Injury Scale; DIC, disseminated intravascular coagulation; ISS, Injury Severity Score; iTBI, isolated traumatic brain injury; SIRS, systemic inflammatory response syndrome; TBI, traumatic brain injury*.

**Table 2:** Requirement for transfusion and emergency surgery, and in-hospital mortality.

	**Non-TBI**	**iTBI**	***p*** **-value**
	***n*** **= 40**	***n*** **= 34**	
Operation within 24 h after admission, *n* (%)	17 (42.5)	14 (42.4)	0.995
Massive transfusion, *n* (%)	2 (5.0)	4 (11.8)	0.404
3-h Transfusion			
Packed red blood cells (mL)	0 (0–0)	0 (0–0)	0.909
Fresh frozen plasma (mL)	0 (0–0)	0 (0–0)	0.636
Platelet concentrate (U)	0 (0–0)	0 (0–0)	1.000
Cryoprecipitate (U)	0 (0–0)	0 (0–0)	1.000
24-h Transfusion			
Packed red blood cells (mL)	0 (0–280)	0 (0–0)	0.772
Fresh frozen plasma (mL)	0 (0–0)	0 (0–480)	0.693
Platelet concentrate (U)	0 (0–0)	0 (0–0)	0.705
Cryoprecipitate (U)	0 (0–0)	0 (0–0)	1.000
In-hospital mortality, *n* (%)	1 (2.5)	4 (12.1)	0.169

*Reported median (interquartile range) for continuous variables*.

*iTBI, isolated traumatic brain injury; TBI, traumatic brain injury*.

### Comparison of Coagulofibrinolytic Changes Between the iTBI and Non-TBI Groups

**Figure 1:**
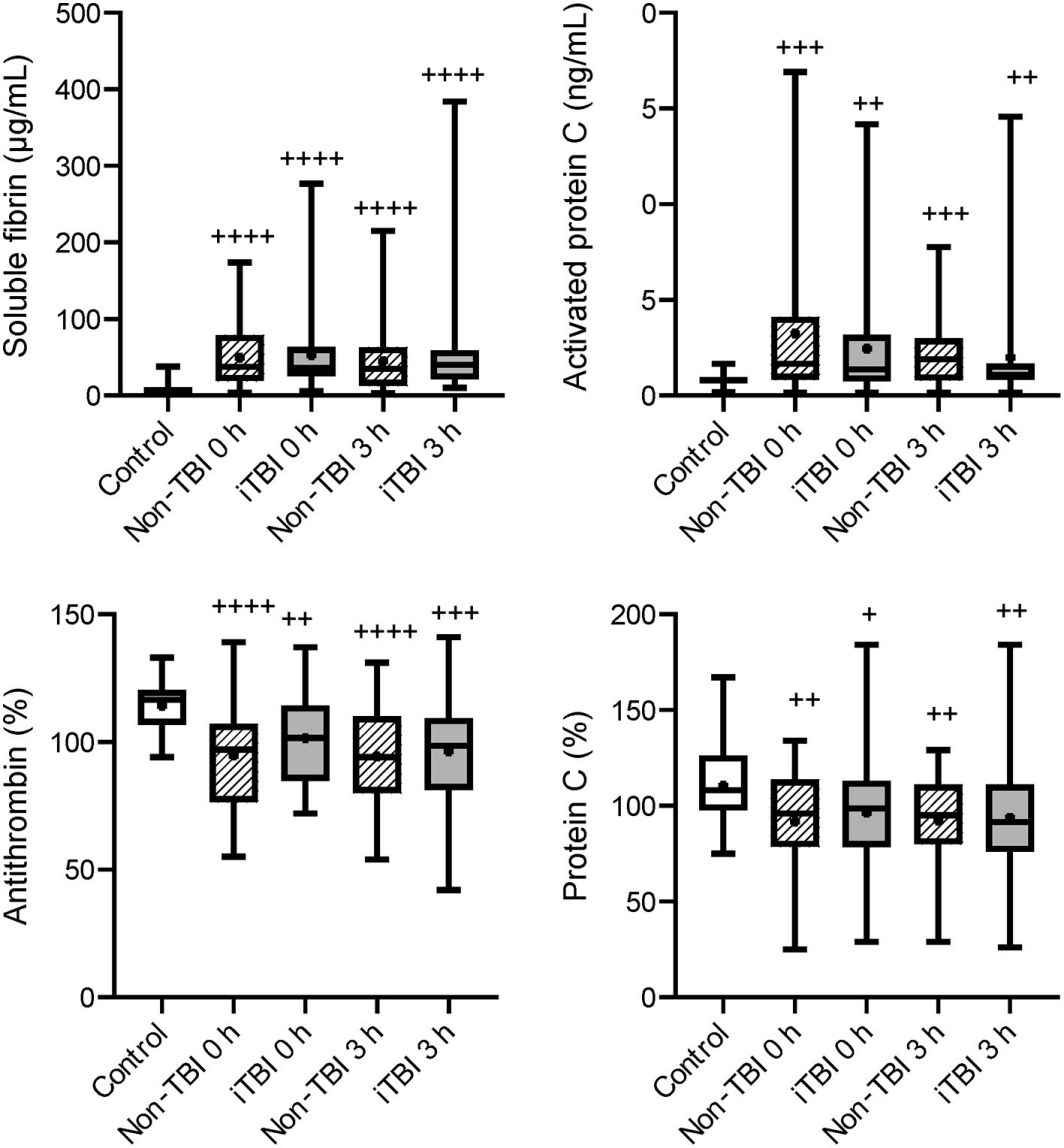
Serial changes in the coagulation-related molecular markers. Healthy controls (white box), non-traumatic brain injury (TBI) (hatched boxes), and isolated TBI (iTBI) (gray boxes) at presentation to the emergency department (0 h) and 3 h after hospital arrival (3 h). The horizontal bars in the box indicate the median (middle) and interquartile ranges (upper 25% and lower 75%). Black boxes are mean values. +*p* < 0.05 vs. healthy controls; ++*p* < 0.01 vs. healthy controls; + + +*p* < 0.001 vs. healthy controls; + + + +*p* < 0.0001 vs. healthy controls. None of the markers significantly differed between the iTBI and non-TBI groups.

**Figure 2:**
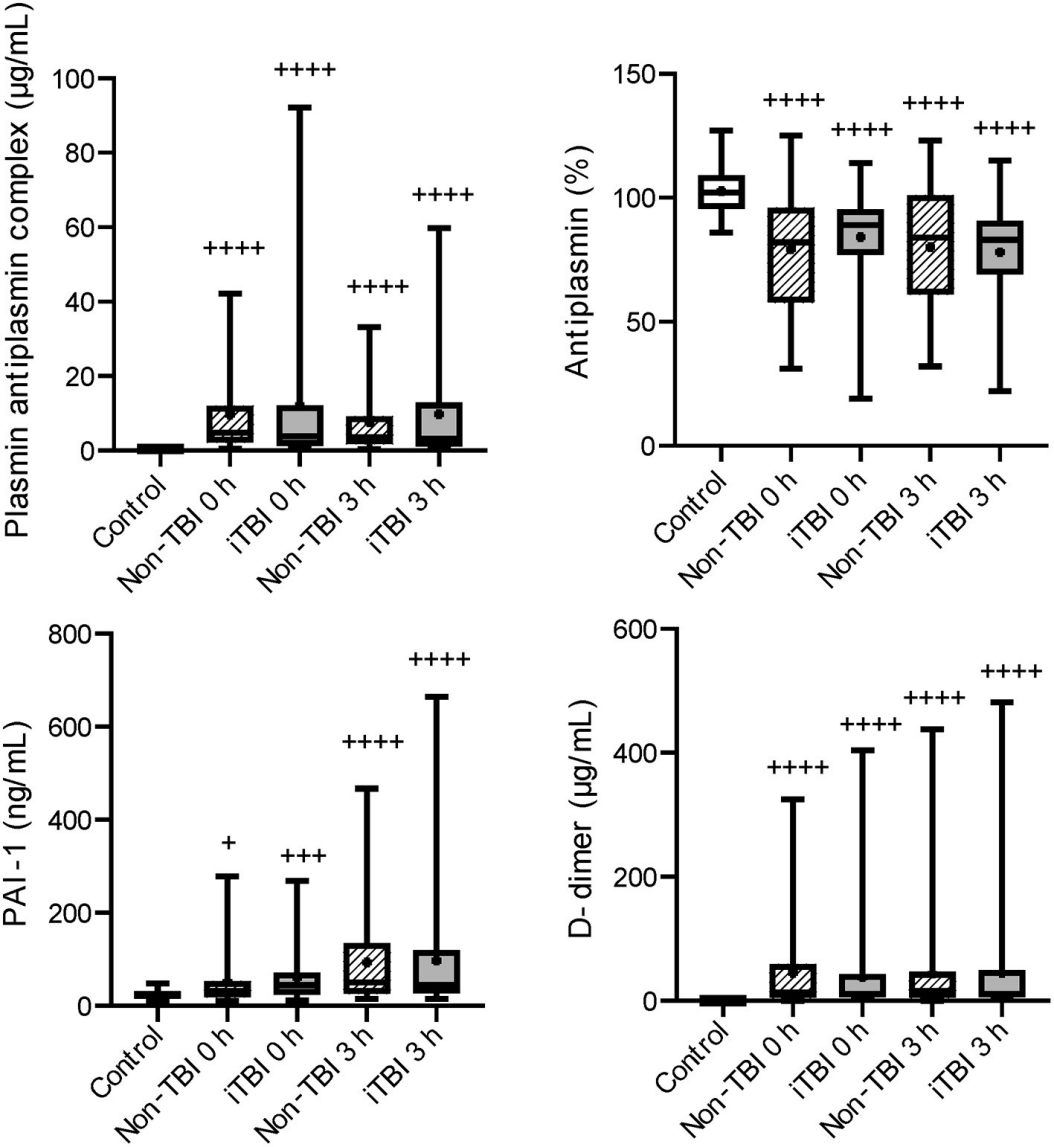
Serial changes in the fibrinolysis-related molecular markers. Healthy controls (white box), non-traumatic brain injury (TBI) (hatched boxes), and isolated TBI (iTBI) (gray boxes) at presentation to the emergency department (0 h) and 3 h after hospital arrival (3 h). Horizontal bars in the box indicate the median (middle) and interquartile ranges (upper 25% and lower 75%). Black boxes are mean values. +*p* < 0.05 vs. healthy controls, + + +*p* < 0.001 vs. healthy controls, + + + +*p* < 0.0001 vs. healthy controls. None of the markers significantly differed between the iTBI and non-TBI groups.

Serial changes in the molecular markers of coagulation are shown in Figure 1. The levels of anticoagulation factors such as antithrombin and protein C were lower, while the levels of soluble fibrin and APC were significantly higher in the iTBI and non-TBI groups than in the healthy control group. These coagulation markers showed similar changes in patients with and without TBI. As shown in Figure 2, increased levels of plasmin-antiplasmin complex, D-dimer, and PAI-1 were observed in iTBI and non-TBI patients than in healthy controls. The levels of antiplasmin significantly decreased in the iTBI and non-TBI groups compared to those in the control group. These fibrinolysis-related molecular markers also underwent similar changes in the iTBI and non-TBI groups. In addition, serial changes in platelet counts and global markers of coagulation and fibrinolysis were similar between the groups (Supplementary Table 2).

### Factors Associated With Lactate Levels

**Figure 3:**
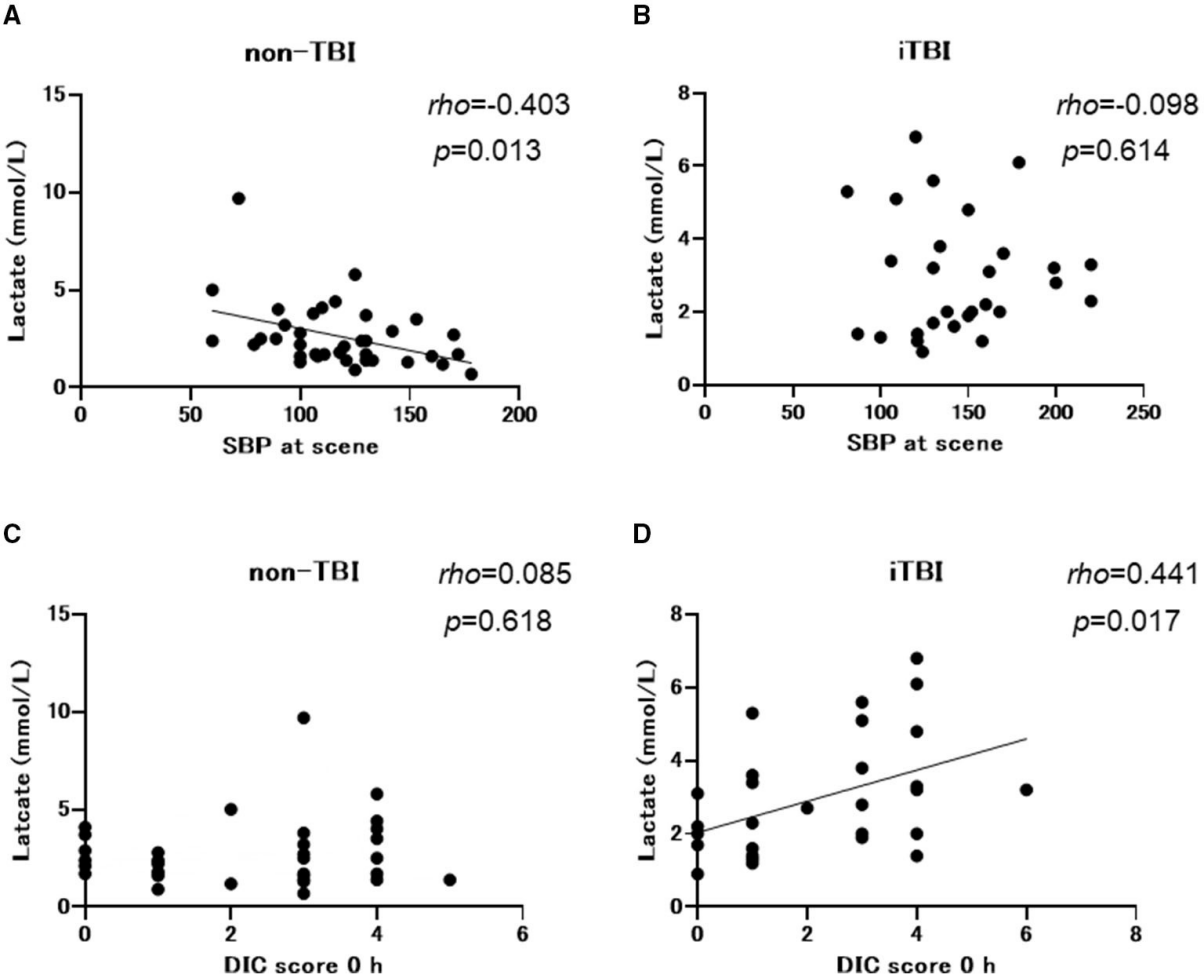
Spearman's rank correlation coefficients between the lactate levels and systolic blood pressure (SBP) at scene and the disseminated intravascular coagulation (DIC) score at arrival to the emergency department. TBI, traumatic brain injury; iTBI, isolated traumatic brain injury.

Non-TBI patients exhibited lower systolic blood pressure at the scene and at the ED with a higher incidence of shock defined as a systolic blood pressure of <90 mmHg at the ED than the iTBI group. However, the incidence of shock defined by lactate levels of >2 mmol/L was identical between the two groups (Table 1). There was a negative correlation between systolic blood pressure and lactate levels in the non-TBI group (Spearman's rho = −0.403, *p* = 0.013; Figure 3A), while no significant correlation was observed between these variables in the iTBI group (Spearman's rho = 0.098, *p* = 0.614; Figure 3B). By contrast, no correlation was observed between lactate levels and the DIC score at 0 h in the non-TBI group (Spearman's rho = 0.085, *p* = 0.618; Figure 3C), but a positive correlation was observed between these values in the iTBI group (Spearman's rho = 0.441, *p* = 0.017; Figure 3D).

### Factors Associated With the Development of DIC in the iTBI Group

Logistic regression analysis was performed to evaluate the factors associated with the development of DIC. Multiple logistic regression analysis indicated that ISS was an independent predictor of DIC development in patients with iTBI (odds ratio=1.237, *p*=0.018; Table 3).

**Table 3:** Results of logistic regression analyses of variables predicting the development of disseminated intravascular coagulation in patients with isolated traumatic brain injury.

	**Univariable**			**Multivariable**		
	**Odds ratio**	***p-*** **value**	**95% CI**	**Odds ratio**	***p-*** **value**	**95% CI**
Age	1.027	0.202	0.986–1.070			
Sex	3.281	0.120	0.733–14.683			
ISS	1.220	0.018	1.034–1.439	1.237	0.018	1.034–1.439
SBP at scene	1.004	0.595	0.989–1.020			
Shock (ABP) at scene	2.769	0.425	0.226–33.879			
SBP at ED	1.025	0.078	0.997–1.053			
Shock (ABP) at ED, *n* (%)	0.000	1.000	0–0			
Lactate 0 h	1.403	0.177	0.858–2.295			
Shock (lac), *n* (%)	3.500	0.172	0.579–21.161			

*Shock (ABP) represents a systolic blood pressure of <90 mmHg. Shock (lac) represents lactate levels of >2 mmol/L at the emergency department (ED)*.

*CI, confidence interval; ED, emergency department; ISS, injury severity score; iTBI, isolated traumatic brain injury; SBP, systolic blood pressure; TBI, traumatic brain injury*.

### Baseline Characteristics, Transfusion, and In-Hospital Mortality in iTBI Patients With and Without DIC

**Figure 4:**
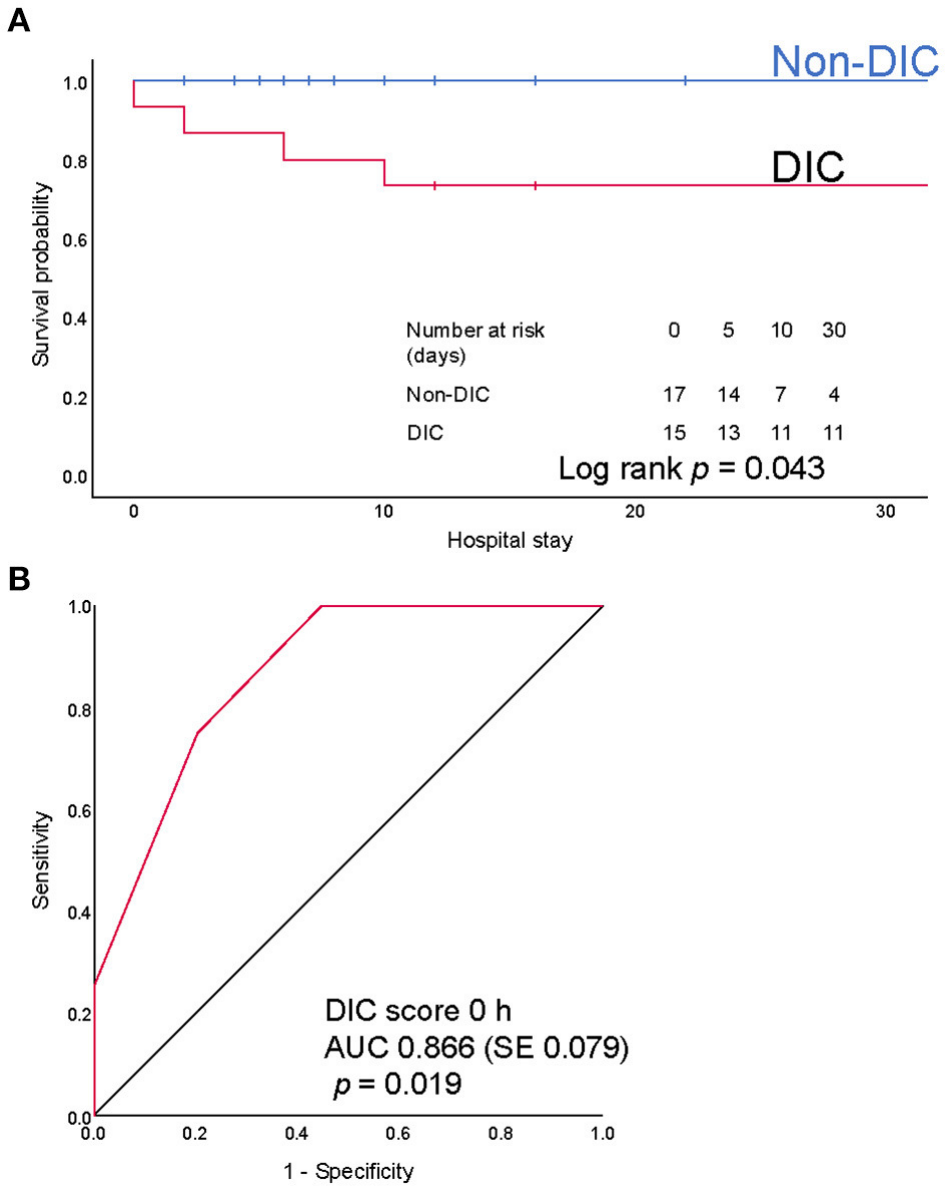
**(A)** Kaplan-Meier survival provability curves for mortality during hospitalization. Numbers at risk represent the number of patients with isolated traumatic brain injury (iTBI) with or without disseminated intravascular coagulation (DIC) at risk of death on the indicated days. **(B)** Receiver operating characteristics (ROC) curves of the DIC score immediately after arrival at emergency department. AUC, area under the ROC curve; SE, standard error.

Next, we focused on iTBI patients and divided them into the DIC group (*n* = 15) and the non-DIC group (*n* = 19). The baseline characteristics of the patients are presented in Table 4. The median ISSs in the DIC and non-DIC groups were 25 and 17, respectively. DIC patients had a higher AIS score in the head and worse Glasgow Coma Scale scores than non-DIC patients. The physiological parameter values at the scene and ED did not differ between the groups. Only few patients had blood pressure-defined shock in both groups, whereas >50% of the patients had lactate-defined shock (DIC, 83.3%; non-DIC, 58.8%). The median lactate levels in the DIC and non-DIC groups were 4.3 and 2.0 mmol/L, respectively. The requirements for massive transfusion and emergency surgery for TBI were significantly higher, and in-hospital mortality was higher in the DIC group than in the non-DIC group (Table 5). The survival probability was significantly lower among iTBI patients diagnosed with DIC immediately after presentation to the ED than among patients without DIC (log rank *p*=0.043; Figure 4A). ROC curves indicated that the DIC scores immediately after arrival to the ED were a good predictor of in-hospital mortality in patients with iTBI (AUC 0.866, *p*=0.019; Figure 4B).

**Table 4:** Demographics and parameters at the scene and admission to the emergency department in isolated traumatic brain injury patients with and without disseminated intravascular coagulation.

	**Non-DIC**	**DIC**	***p*** **-value**
	***n*** **= 19**	***n*** **= 15**	
Demographics			
Age (years)	59 (51–73)	58 (49–74)	0.242
Male sex, *n* (%)	15 (78.9)	8 (53.3)	0.112
Charlson comorbidity index	0 (0–0)	0 (0–1)	0.190
Glasgow coma scale	14 (13–15)	7 (3–9)	0.004
ISS	17 (16–17)	25 (16–25)	0.120
AIS			
Head	4 (4–4)	5 (4–5)	0.004
Face	0 (0–0)	0 (0–0)	0.023
Neck	0 (0–0)	0 (0–0)	0.451
Thorax	0 (0–0)	0 (0–0)	1.000
Abdomen	0 (0–0)	0 (0–0)	0.811
Spine			
Cervical	0 (0–0)	0 (0–0)	1.000
Thoracic	0 (0–0)	0 (0–0)	1.000
Lumber	0 (0–0)	0 (0–0)	1.000
Upper extremity	0 (0–0)	0 (0–0)	0.811
Lower extremity	0 (0–0)	0 (0–0)	1.000
External	0 (0–0)	0 (0–0)	0.918
SIRS criteria	1 (1–2)	3 (1–3)	0.116
Shock (ABP) at scene, *n* (%)	1 (5.3)	2 (13.3)	0.409
Shock (ABP) at ED, *n* (%)	1 (5.3)	0 (0)	0.559
Shock (lac), *n* (%)	10 (58.8)	10 (83.3)	0.160
At the scene			
Systolic blood pressure (mmHg)	138 (121–155)	144 (138–179)	0.537
Diastolic blood pressure (mmHg)	78 (60–86)	83 (70–109)	0.708
Heart rate (bpm)	84 (77–87)	80 (66–98)	0.421
Respiratory rate (breaths/min)	18 (18–21)	18 (16–18)	0.167
At the ED			
Systolic blood pressure (mmHg)	138 (128–155)	144 (138–179)	0.083
Diastolic blood pressure (mmHg)	82 (65–90)	84 (74–98)	0.202
Heart rate (bpm)	74 (70–86)	95 (81–98)	0.401
Respiratory rate (breaths/min)	18 (16–19)	17 (15–20)	0.319
Lactate (mmol/L)	2.0 (1.3–2.9)	4.3 (3.2–6.1)	0.152
Body temperature (°C)	36.8 (36.0–37.0)	36.0 (35.7–36.8)	0.132

*Reported proportions (counts) for categorical variables and medians (interquartile ranges) for continuous variables. Shock (ABP) represents a systolic blood pressure of <90 mmHg. Shock (lac) represents lactate levels of >2 mmol/L in the emergency department (ED)*.

*AIS, Abbreviated Injury Scale; DIC, disseminated intravascular coagulation; ISS, Injury Severity Score; iTBI, isolated traumatic brain injury; SIRS, systemic inflammatory response syndrome*.

**Table 5:** Requirement for transfusion and emergency surgery, and in-hospital mortality in isolated traumatic brain injury patients with and without disseminated intravascular coagulation.

	**Non-DIC**	**DIC**	***p*** **-value**
	***n*** **= 19**	***n*** **= 15**	
Operation for TBI within 24 h after admission, *n* (%)	4 (21.1)	9 (60.0)	0.024
Massive transfusion, *n* (%)	0 (0)	4 (26.7)	0.029
3-h transfusion			
Packed red blood cells (mL)	0 (0–0)	0 (0–0)	0.451
Fresh frozen plasma (mL)	0 (0–0)	0 (0–0)	0.681
Platelet concentrate (U)	0 (0–0)	0 (0–0)	1.000
Cryoprecipitate (U)	0 (0–0)	0 (0–0)	1.000
24-h transfusion			
Packed red blood cells (mL)	0 (0–0)	560 (0–5,880)	0.096
Fresh frozen plasma (mL)	0 (0–0)	1,080 (480–8,160)	0.077
Platelet concentrate (U)	0 (0–0)	0 (0–32)	0.202
Cryoprecipitate (U)	0 (0–0)	0 (0–0)	1.000
In-hospital mortality, *n* (%)	0 (0)	4 (26.7)	0.033

*Reported median (interquartile range) for continuous variables*.

*TBI, traumatic brain injury; DIC, disseminated intravascular coagulation*.

### Comparison of Coagulofibrinolytic Changes Between iTBI Patients With and Without DIC

**Figure 5:**
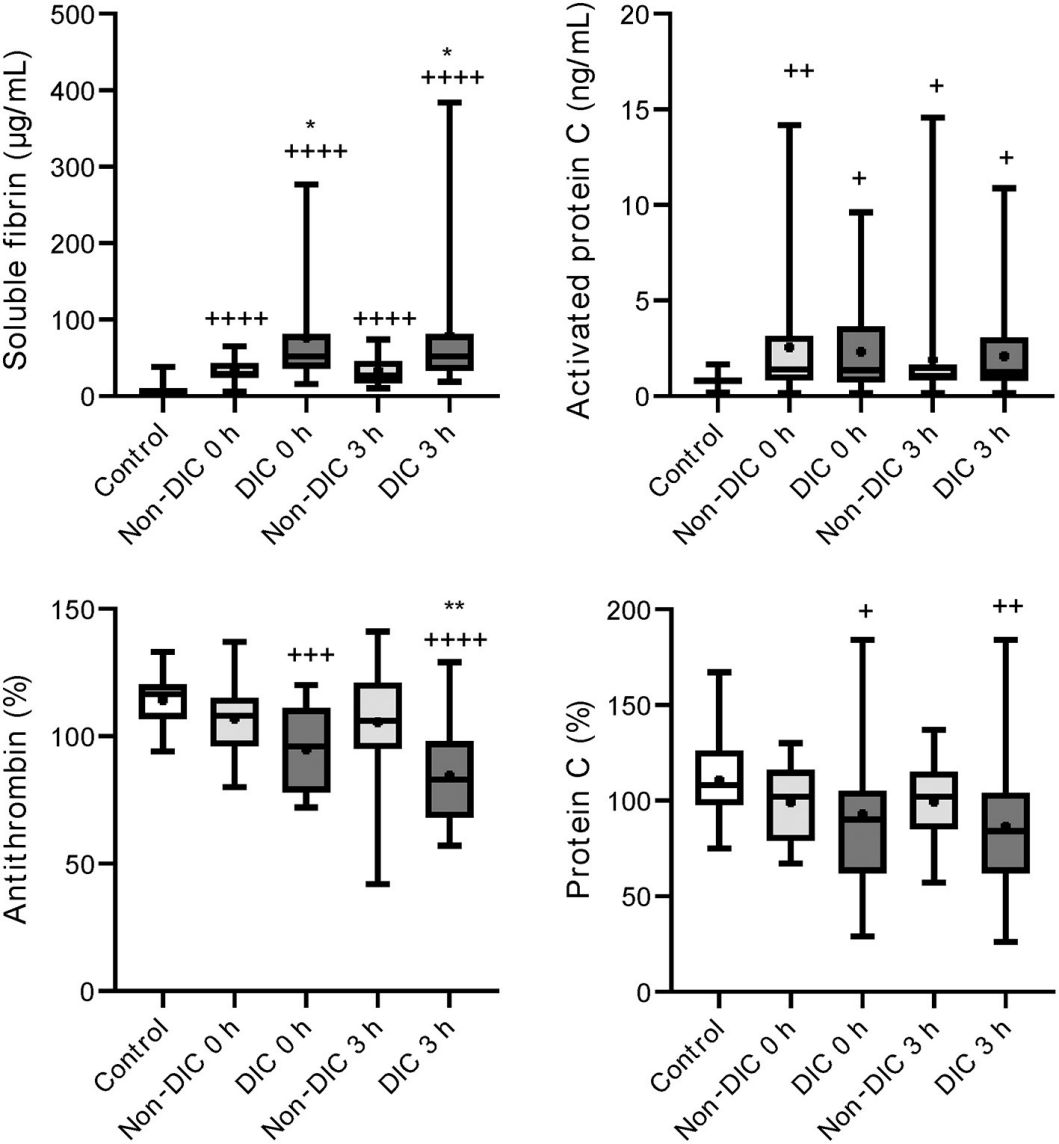
Serial changes in the coagulation-related molecular markers in patients with isolated traumatic brain injury. Healthy controls (white box), non-disseminated intravascular coagulation (DIC) (light gray boxes), and DIC (dark gray boxes) at presentation to the emergency department (0 h) and 3 h after hospital arrival (3 h). Horizontal bars in the box indicate the median (middle) and interquartile ranges (upper 25% and lower 75%). Black boxes represent the mean values. +*p* < 0.05 vs. healthy controls, ++*p* < 0.01 vs. healthy controls, + + +*p* < 0.001 vs. healthy controls, + + + +*p* < 0.0001 vs. healthy controls, ^*^p < 0.05 vs. non-DIC, ^**^p < 0.01 vs. non-DIC.

**Figure 6:**
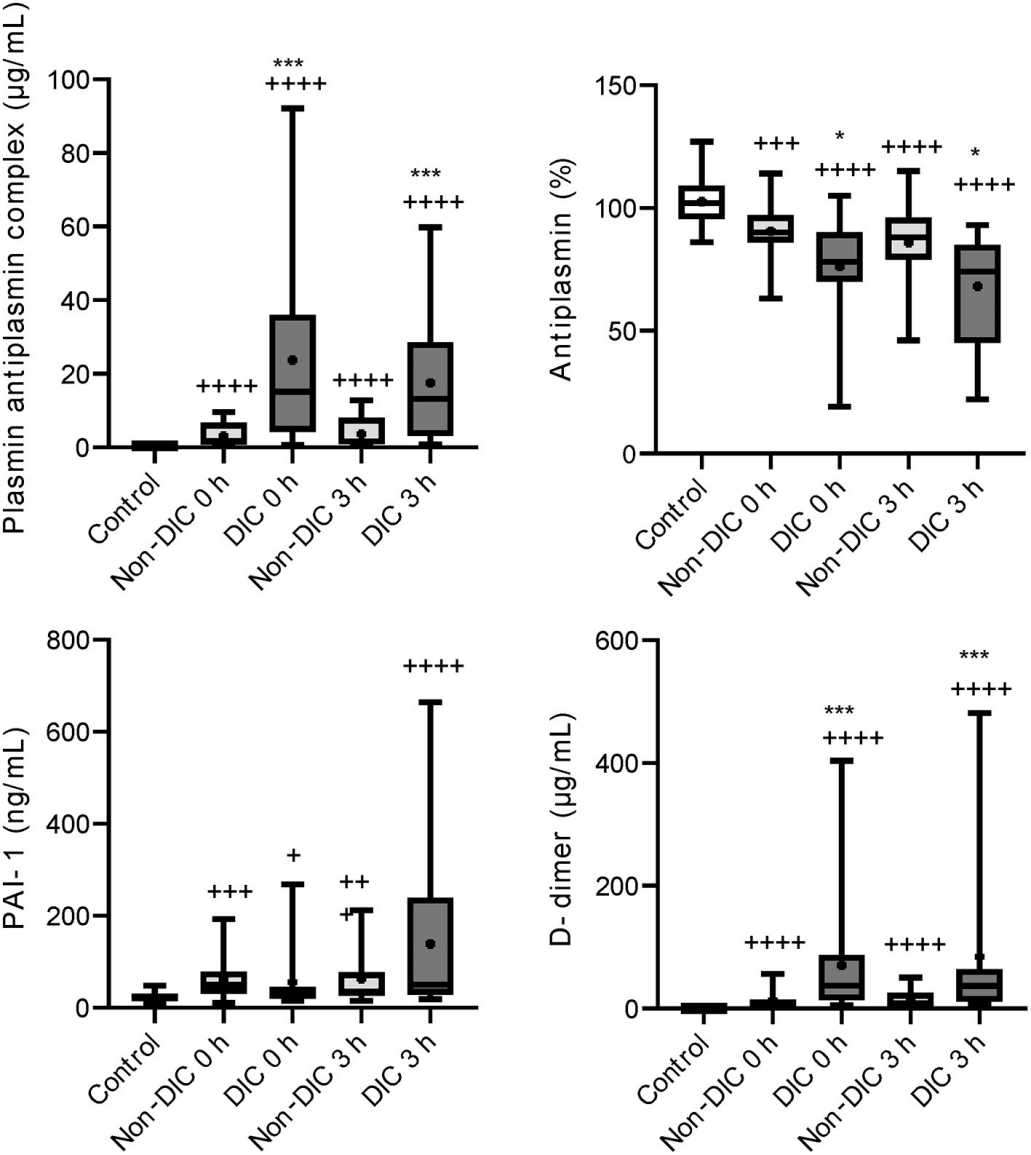
Serial changes in the fibrinolysis-related molecular markers in patients with isolated traumatic brain injury. Healthy controls (white box), non-disseminated intravascular coagulation (DIC) (light gray boxes) and DIC (dark gray boxes) at presentation to the emergency department (0 h) and 3 h after hospital arrival (3 h). Horizontal bars in the box indicate the median (middle) and interquartile ranges (upper 25% and lower 75%). Black boxes represent the mean values. +*p* < 0.005 vs. healthy controls, ++*p* < 0.001 vs. healthy controls, + + +*p* < 0.001 vs. healthy controls, + + ++*p* < 0.0001 vs. healthy controls, ^*^p < 0.05 vs. non-DIC, ^***^p < 0.001 vs. non-DIC.

Higher levels of soluble fibrin and lower levels of antithrombin were confirmed in iTBI patients with DIC than in those without DIC (Figure 5). As shown in Figure 6, there were increased plasmin generation and fibrinolysis and lower levels of antiplasmin at 0 and 3 h in the iTBI with DIC group than in the iTBI without DIC group. The PAI-1 levels in iTBI patients with DIC at 3 h after hospital arrival tended to be higher than those in patients without DIC (*p* = 0.091). Higher levels of FDP at 0 h and lower platelet counts were observed in iTBI patients with DIC than in those without DIC (Supplementary Table 3).

## Discussion

This study aimed to elucidate the pathophysiology of TBI-induced coagulopathy by comparing the coagulofibrinolytic changes in iTBI patient with those in non-TBI trauma patients, to determine the associated factors, and to identify the clinical significance of DIC diagnosis in patients with iTBI. In the present study, similar requirements for massive transfusion and in-hospital mortality were observed between iTBI and non-TBI patients with a similar severity of trauma. The changes in the levels of coagulofibrinolytic biomarkers were also identical between the groups. The lactate levels in the iTBI group were positively correlated with DIC scores but not with blood pressure. Moreover, the ISS was an independent predictor of DIC development in patients with iTBI. iTBI patients with DIC showed a significantly higher requirement for emergency surgery for TBI, and a higher incidence of massive transfusion than did those without DIC. DIC development was significantly associated with poor hospital survival, and DIC scores at 0 h were predictive of in-hospital mortality. In patients with iTBI with DIC, marked thrombin and plasmin generation was confirmed.

Although numerous mechanisms that are potentially associated with coagulopathy after TBI have been suggested, we previously demonstrated that the main pathomechanism of TIC, with or without TBI, is DIC (19–21). DIC is characterized by increased thrombin generation and subsequent consumption coagulopathy due to damage-associated molecular patterns derived from injured cells and tissues (22). That is, DIC is caused by trauma itself (23). However, another theory advocates that TBI alone does not cause early coagulopathy (24) and that coagulopathy occurs only in patients with shock-induced profound acidosis and high lactate concentrations (25–27). The results of the present study indicated no correlation between lactate levels and systolic blood pressure (Figure 3B) in iTBI patients, but the positive correlation between lactate levels and DIC score (Figure 3D) in those patients implies that the increase in lactate levels in TBI patients is caused by DIC-mediated secondary tissue hypoperfusion rather than by hypotension due to hemorrhagic shock. The results demonstrated that ISS was an independent predictor of pathological coagulofibrinolytic changes, namely DIC, in iTBI patients (Table 2), which also supports the concept that TIC is caused by trauma itself, with or without shock (23). The current results revealed similar coagulofibrinolytic changes, characterized by DIC with hyperfibrinolysis, between iTBI and non-TBI (Figures 1, 2), which was in line with the findings of a previous study (28).

iTBI patients with DIC had a higher requirement for massive transfusion and emergency surgery and had a higher in-hospital mortality than did those without DIC (Table 5). In addition, the DIC score in the iTBI group immediately after arrival to the hospital was predictive of in-hospital mortality (Figure 4B). Similar results have been reported not only in the setting of iTBI (2, 3), but also in other trauma settings (29). These results suggest the importance of DIC diagnosis in severely injured patients with or without TBI.

The present study revealed marked thrombin generation with lower levels of antithrombin in iTBI patients, particularly those with DIC (Figure 5). Previous studies confirmed such thrombin generation after iTBI, as evaluated by the high levels of the thrombin–antithrombin complex, prothrombin fragment 1 + 2, fibrinopeptide A, and fibrin monomers (28, 30, 31). High levels of soluble tissue factors have also been found (30, 32). Injury to the blood–brain barrier, caused by direct cerebral vascular disruption, potentially releases brain tissue factors into systemic circulation (5, 33). Tissue factors exposed to blood bind extensively to factor VIIa, followed by the initiation of the extrinsic coagulation pathway and subsequent consumption coagulopathy (23). Massive thrombin generation due to the activation of coagulation may lead to the consumption of anticoagulant factors, such as antithrombin and protein C, followed by further activation of coagulation. These changes contribute to hypercoagulability inside the vessels, leading to intravascular microthrombosis in the brain and other organs, and hypocoagulability outside the vessels due to consumptive coagulopathy, leading to the progression of intracranial hemorrhage (34).

This study demonstrated marked plasmin generation with lower levels of antiplasmin in iTBI and these changes were prominent in iTBI patients with DIC (Figure 6). Hyperfibrinolysis after trauma is caused by the release of tissue-type plasminogen activator (t–PA) from the endothelial Weibel-Palade bodies due to traumatic shock-induced tissue hypoperfusion (22). However, patients with iTBI with high lactate levels had no low blood pressure (Figure 3B), and similar results were reported previously (35). These results indicate that increases in lactate levels are not caused by low blood pressure-related hypoperfusion, but by DIC-induced tissue-hypoperfusion. That is, high lactate levels in iTBI with coagulopathy are a result rather than a cause of TIC. Instead of traumatic shock-induced tissue hypoperfusion, the direct release of t-PA from injured brain tissue has been acknowledged as another explanation of hyperfibrinolysis in patients with iTBI (11). A previous study demonstrated that endogenous t-PA increases the lysis of plasma clots and contributes to intracerebral hemorrhage after TBI (36).

## Study Limitations

Several limitations of our study need to be considered. First, although the present dataset was prospectively collected, causal relationships could not be defined because of the retrospective study design and missing values. Second, this study included a small number of patients. Third, we did not distinguish types of TBI (e.g., acute subdural hematoma, traumatic subarachnoid hemorrhage, and contusion), which may affect the pathology and severity of TBI-induced coagulopathy. Fourth, although more patients received tranexamic acid in the iTBI group than in the non-TBI group, the effects of tranexamic acid on the levels of coagulation and fibrinolysis-related markers were not determined. Fifth, bias may exist in trauma types in the non-TBI group, as suggested by the finding that many patients in this group had severe thoracic injury. Finally, this was a single nationwide study conducted in a developed country, which may have limited the global generalization of the results.

## Conclusions

The present study demonstrated similar coagulofibrinolytic changes between iTBI and non-TBI patients, which are consistent with DIC. Marked generation of thrombin and plasmin was confirmed in iTBI patients, and these changes were more prominent in iTBI patients with DIC. The development of DIC in patients with iTBI was associated with a poor survival outcome, and DIC scores immediately after hospital arrival could predict in-hospital death. Therefore, the diagnosis of DIC in the early phase of trauma is important for predicting the outcome of severely injured patients, including those with iTBI.

## Data Availability Statement

The raw data supporting the conclusions of this article will be made available by the authors, without undue reservation.

## Ethics Statement

This study was approved under the condition that written informed consent was obtained from the patient or next of kin by the JAAM and the Ethics Committee of each hospital (JAAM, 2014-01; Hokkaido University Graduate School of Medicine, Head institute of the FORECAST group, 014-0307). Written informed consent to participate in this study was provided by the participants' legal guardian/next of kin.

## Author Contributions

TW analyzed the study results, interpreted the data, and drafted the manuscript. AS checked the statistical methods and results. AS, SG, KY, SFujis, DS, SK, HO, TA, TM, JS, JK, NT, RT, KT, SS, YSh, TN, KO, YSa, AH, SFujim, YU, and YO planned the study, decided the methods, developed a web-based registration system, discussed the results, and critically revised the manuscript. All authors have read and approved the final version of the manuscript.

## Funding

This study was supported by the Japanese Association for Acute Medicine (JAAM, 2014-01). This work was also supported in part by JSPS KAKENHI (Grant-in-Aid (C) 2020, 20K09260).

## Conflict of Interest

AS reported receiving personal fees from CSL Behring outside of the submitted work. SG reported receiving personal fees from Asahi Kasei Pharma America Inc. and Asahi Kasei Pharma Japan Inc. outside of the submitted work. The remaining authors declare that the research was conducted in the absence of any commercial or financial relationships that could be construed as a potential conflict of interest.

## Publisher's Note

All claims expressed in this article are solely those of the authors and do not necessarily represent those of their affiliated organizations, or those of the publisher, the editors and the reviewers. Any product that may be evaluated in this article, or claim that may be made by its manufacturer, is not guaranteed or endorsed by the publisher.

## References

[1] Iaccarino C Carretta A Nicolosi F Morselli C. Epidemiology of severe traumatic brain injury. J Neurosurg Sci. (2018) 62:535–41. 10.23736/S0390-5616.18.04532-0 30182649

[2] Harhangi BS Kompanje EJ Leebeek FW Maas AI. Coagulation disorders after traumatic brain injury. Acta Neurochir. (2008) 150:165–75; discussion 75. 10.1007/s00701-007-1475-8 18166989

[3] Epstein DS Mitra B O'Reilly G Rosenfeld JV Cameron PA. Acute traumatic coagulopathy in the setting of isolated traumatic brain injury: a systematic review and meta-analysis. Injury. (2014) 45:819–24. 10.1016/j.injury.2014.01.011 24529718

[4] Zou Z Li L Schäfer N Huang Q Maegele M Gu Z. Endothelial glycocalyx in traumatic brain injury associated coagulopathy: potential mechanisms and impact. J Neuroinflammation. (2021) 18:134. 10.1186/s12974-021-02192-1 34126995 PMC8204552

[5] Maegele M Schöchl H Menovsky T Maréchal H Marklund N Buki A. Coagulopathy and haemorrhagic progression in traumatic brain injury: advances in mechanisms, diagnosis, and management. Lancet Neurol. (2017) 16:630–47. 10.1016/S1474-4422(17)30197-7 28721927

[6] Stein SC Young GS Talucci RC Greenbaum BH Ross SE. Delayed brain injury after head trauma: significance of coagulopathy. Neurosurgery. (1992) 30:160–5. 10.1097/00006123-199202000-00002 1545882

[7] Stein SC Chen XH Sinson GP Smith DH. Intravascular coagulation: a major secondary insult in nonfatal traumatic brain injury. J Neurosurg. (2002) 97:1373–7. 10.3171/jns.2002.97.6.1373 12507136

[8] Stein SC Graham DI Chen XH Smith DH. Association between intravascular microthrombosis and cerebral ischemia in traumatic brain injury. Neurosurgery. (2004) 54:687–91; discussion 91. 10.1227/01.NEU.0000108641.98845.88 15028145

[9] Gando S Sawamura A Hayakawa M. Trauma, shock, and disseminated intravascular coagulation: lessons from the classical literature. Ann Surg. (2011) 254:10–9. 10.1097/SLA.0b013e31821221b1 21368657

[10] Taylor FB Jr Toh CH Hoots WK Wada H Levi M. Towards definition, clinical and laboratory criteria, and a scoring system for disseminated intravascular coagulation. Thromb Haemost. (2001) 86:1327–30. 10.1055/s-0037-1616068 11816725

[11] Gando S. Hemostasis and thrombosis in trauma patients. Semin Thromb Hemost. (2015) 41:26–34. 10.1055/s-0034-1398378 25602698

[12] Kaufman HH Hui KS Mattson JC Borit A Childs TL Hoots WK. Clinicopathological correlations of disseminated intravascular coagulation in patients with head injury. Neurosurgery. (1984) 15:34–42. 10.1097/00006123-198407000-00007 6472592

[13] Stein SC Smith DH. Coagulopathy in traumatic brain injury. Neurocrit Care. (2004) 1:479–88. 10.1385/NCC:1:4:479 16174954

[14] Moore HB Gando S Iba T Kim PY Yeh CH Brohi K. Defining trauma-induced coagulopathy with respect to future implications for patient management: communication from the SSC of the ISTH. J Thromb Haemost. (2020) 18:740–7. 10.1111/jth.14690 32112533

[15] Gando S Shiraishi A Wada T Yamakawa K Fujishima S Saitoh D. A multicenter prospective validation study on disseminated intravascular coagulation in trauma-induced coagulopathy. J Thromb Haemost. (2020) 18:2232–44. 10.1111/jth.14931 32480432

[16] Gando S Saitoh D Ogura H Mayumi T Koseki K Ikeda T. Natural history of disseminated intravascular coagulation diagnosed based on the newly established diagnostic criteria for critically ill patients: results of a multicenter, prospective survey. Crit Care Med. (2008) 36:145–50. 10.1097/01.CCM.0000295317.97245.2D 18090367

[17] American college of chest physicians/society of critical care medicine consensus conference: definitions for sepsis and organ failure and guidelines for the use of innovative therapies in sepsis. Crit Care Med. (1992) 20:864–74. 10.1097/00003246-199206000-00025 1597042

[18] Charlson ME Pompei P Ales KL MacKenzie CR. A new method of classifying prognostic comorbidity in longitudinal studies: development and validation. J Chronic Dis. (1987) 40:373–83. 10.1016/0021-9681(87)90171-8 3558716

[19] Gando S Nanzaki S Kemmotsu O. Disseminated intravascular coagulation and sustained systemic inflammatory response syndrome predict organ dysfunctions after trauma: application of clinical decision analysis. Ann Surg. (1999) 229:121–7. 10.1097/00000658-199901000-00016 9923809 PMC1191617

[20] Gando S. Disseminated intravascular coagulation in trauma patients. Semin Thromb Hemost. (2001) 27:585–92. 10.1055/s-2001-18864 11740682

[21] Gando S. Acute coagulopathy of trauma shock and coagulopathy of trauma: a rebuttal. You are now going down the wrong path. J Trauma. (2009) 67:381–3. 10.1097/TA.0b013e3181a84f63 19667894

[22] Gando S Otomo Y. Local hemostasis, immunothrombosis, and systemic disseminated intravascular coagulation in trauma and traumatic shock. Crit Care. (2015) 19:72. 10.1186/s13054-015-0735-x 25886801 PMC4337317

[23] Gando S Hayakawa M. Pathophysiology of trauma-induced coagulopathy and management of critical bleeding requiring massive transfusion. Semin Thromb Hemost. (2016) 42:155–65. 10.1055/s-0035-1564831 26716498

[24] Cohen MJ Brohi K Ganter MT Manley GT Mackersie RC Pittet JF. Early coagulopathy after traumatic brain injury: the role of hypoperfusion and the protein C pathway. J Trauma. (2007) 63:1254–61; discussion 61–2. 10.1097/TA.0b013e318156ee4c 18212647

[25] Brohi K Cohen MJ Ganter MT Matthay MA Mackersie RC Pittet JF. Acute traumatic coagulopathy: initiated by hypoperfusion: modulated through the protein C pathway? Ann Surg. (2007) 245:812–8. 10.1097/01.sla.0000256862.79374.31 17457176 PMC1877079

[26] Brohi K Cohen MJ Ganter MT Schultz MJ Levi M Mackersie RC. Acute coagulopathy of trauma: hypoperfusion induces systemic anticoagulation and hyperfibrinolysis. J Trauma. (2008) 64:1211–7; discussion 7. 10.1097/TA.0b013e318169cd3c 18469643

[27] Hess JR Brohi K Dutton RP Hauser CJ Holcomb JB Kluger Y. The coagulopathy of trauma: a review of mechanisms. J Trauma. (2008) 65:748–54. 10.1097/TA.0b013e3181877a9c 18849786

[28] Gando S Nanzaki S Kemmotsu O. Coagulofibrinolytic changes after isolated head injury are not different from those in trauma patients without head injury. J Trauma. (1999) 46:1070–6; discussion 6–7. 10.1097/00005373-199906000-00018 10372628

[29] Wada T Shiraishi A Gando S Yamakawa K Fujishima S Saitoh D. Disseminated intravascular coagulation immediately after trauma predicts a poor prognosis in severely injured patients. Sci Rep. (2021) 11:11031. 10.21203/rs.3.rs-199070/v1 34040091 PMC8154895

[30] Di Battista AP Rizoli SB Lejnieks B Min A Shiu MY Peng HT. Sympathoadrenal activation is associated with acute traumatic coagulopathy and endotheliopathy in isolated brain injury. Shock. (2016) 46:96–103. 10.1097/SHK.0000000000000642 27206278 PMC4978599

[31] Esnault P Mathais Q D'Aranda E Montcriol A Cardinale M Cungi PJ. Ability of fibrin monomers to predict progressive hemorrhagic injury in patients with severe traumatic brain injury. Neurocrit Care. (2020) 33:182–95. 10.1007/s12028-019-00882-6 31797276

[32] Rhind SG Crnko NT Baker AJ Morrison LJ Shek PN Scarpelini S. Prehospital resuscitation with hypertonic saline-dextran modulates inflammatory, coagulation and endothelial activation marker profiles in severe traumatic brain injured patients. J Neuroinflammation. (2010) 7:5. 10.1186/1742-2094-7-5 20082712 PMC2819256

[33] Maegele M Aversa J Marsee MK McCauley R Chitta SH Vyakaranam S. Changes in coagulation following brain injury. Semin Thromb Hemost. (2020) 46:155–66. 10.1055/s-0040-1702178 32160642

[34] Wada T Yamakawa K. Trauma-induced coagulopathy: the past, present, and future: a comment. J Thromb Haemost. (2019) 17:1571–4. 10.1111/jth.14571 31479182

[35] Wada T Gando S Maekaw K Katabami K Sageshima H Hayakawa M. Disseminated intravascular coagulation with increased fibrinolysis during the early phase of isolated traumatic brain injury. Crit Care. (2017) 21:219. 10.1186/s13054-017-1808-9 28826407 PMC5568862

[36] Hijazi N Abu Fanne R Abramovitch R Yarovoi S Higazi M Abdeen S. Endogenous plasminogen activators mediate progressive intracerebral hemorrhage after traumatic brain injury in mice. Blood. (2015) 125:2558–67. 10.1182/blood-2014-08-588442 25673638 PMC4400292

