# Supplementary material for: Pathophysiology of Coagulopathy Induced by Traumatic Brain Injury Is Identical to That of Disseminated Intravascular Coagulation With Hyperfibrinolysis
Source: Front Med (Lausanne). 2021 Nov 15;8:767637. doi: 10.3389/fmed.2021.767637 (PMC8634586; doi:10.3389/fmed.2021.767637)

Supplementary Material

**Supplementary Table 1. Scoring system for disseminated intravascular coagulation (DIC) by the Japanese Association for Acute Medicine**

----------------------------------------------------------------------------------------------------------------

1. Clinical conditions that may be associated with DIC

1) Sepsis/severe infection (any micro-organism)

2) Trauma/burn/surgery

3) Vascular abnormalities

- large vascular aneurysms

- giant hemangioma

- vasculitis

4) Severe toxic or immunological reactions

- snakebite

- recreational drugs

- transfusion reactions

- transplant rejection

5) Malignancy (with the exception of bone marrow suppression)

6) Obstetric calamities

7) Conditions that may be associated with SIRS

- organ destruction (e.g. severe pancreatitis)

- severe hepatic failure

- ischemia/hypoxia/shock

- heat stroke/malignant syndrome

- fat embolism

- rhabdomyolysis

- other

8) Other

-----------------------------------------------------------------------------------------------------------

2. Clinical conditions that should be carefully ruled out

A. Thrombocytopenia

1) dilution and abnormal distribution

massive blood loss and transfusion, massive infusion

2) increased platelet destruction

ITP, TTP/HUS, HIT, drugs, viral infection, alloimmune destruction, APS, HELLP, extracorporeal circulation

3) decreased platelet production

viral infection, drugs, radiation, nutritional deficiency (vitamin B12, folic acid), disorders of hematopoiesis, liver disease, HPS

4) spurious decrease

EDTA-dependent agglutinins, insufficient anticoagulation of blood samples

5) other

hypothermia, artificial devices in the vessel

B. Prolonged prothrombin time

anticoagulation therapy, anticoagulant in blood samples, vitamin K deficiency, liver cirrhosis, massive blood loss and transfusion

C. Elevated FDP

Thrombosis, hemostasis and wound healing, hematoma, pleural effusion, ascites, anticoagulant in blood samples, antifibrinolytic therapy

D. Other

---------------------------------------------------------------------------------------------------------------

3. The diagnostic algorithm for SIRS

1) temperature > 38°C or < 36 °C

2) heart rate > 90 bpm

3) respiratory rate > 20 breaths/min or PaCO_2_ < 32 torr (< 4.3 kPa)

4) white blood cell count > 12,000 cells/mm^3^, < 4,000 cells/mm^3^, or 10% immature (band) forms

---------------------------------------------------------------------------------------------------------------

4. The diagnostic algorithm

Score

SIRS criteria

≥3 1

0-2 0

Platelet counts (10^9^/L)

<80 or greater than 50% decrease within 24 h 3

≥80 <120 or greater than 30% decrease within 24 h 1

≥120 0

Prothrombin time (value of patient/normal value)

≥1.2 1

<1.2 0

Fibrin/fibrinogen degradation products (mg/L)

≥25 3

≥10 <25 1

<10 0

Diagnosis

Four points or more DIC

-----------------------------------------------------------------------------------------------------------------

SIRS, systemic inflammatory response syndrome; ITP, idiopathic thrombocytopenic purpura; TTP, thrombotic thrombocytopenic purpura; HUS, hemolytic uremic syndrome; HIT, heparin-induced thrombocytopenia; APS, antiphospholipid syndrome; HELLP, hemolysis, elevated liver enzymes, and low platelet; HPS, hemophagocytic syndrome; EDTA, ethylenediaminetetraacetic acid; FDP, fibrin/fibrinogen degradation products.

**Supplementary Table 2. Platelet counts, global markers of coagulation and fibrinolysis in patients with isolated traumatic brain injury patients and non-traumatic brain injury patients**

|  | 0h | 3h | 24h |
| --- | --- | --- | --- |
| Platelet counts (10^9^/L) |  |  |  |
| Non-TBI | 211(177-272） | 189(151-246) | 167(121-204) |
| iTBI | 208(195-240) | 193(120-223) | 183(140-200) |
| Prothrombin time (INR) |  |  |  |
| Non-TBI | 1.01(.098-1.10) | 1.05(1.00-1.15) | 1.10(1.01-1.14) |
| iTBI | 1.00(0.95-1.05) | 1.07(0.96-1.11) | 1.07(1.03-1.11) |
| APTT (sec) |  |  |  |
| Non-TBI | 26.9(24.4-28.5) | 27.7(26.1-33.7) | 30.4(28.8-33.4) |
| iTBI | 25.7(23.8-27.7) | 28.4(25.0-31.3) | 29.7(27.2-32.9) |
| Fibrinogen (g/L) |  |  |  |
| Non-TBI | 2.65(2.17-3.10) | 2.35(1.77-3.09) | 3.19(2.67-4.17) |
| iTBI | 2.38(2.24-2.85) | 2.15(1.60-2.74) | 3.04(2.34-3.90) |
| FDP (mg/L) |  |  |  |
| Non-TBI | 23.0(9.5-88.5) | 31.0(9.1-106) | 14.8(4.6-40.5) |
| iTBI | 22.2(14.8-55.2) | 21.3(8.0-94.6) | 6.4(3.3-31.5) |
| FDP/D-dimer ratio |  |  |  |
| Non-TBI | 2.26(1.56-2.88) | 2.03(1.81-2.62) | 2.30(2.09-2.60) |
| iTBI | 2.31(1.60-3.16) | 2.03(1.52-2.80) | 2.13(1.81-2.71) |

APTT, activated partial thromboplastin time; DIC, disseminated intravascular coagulation; FDP, fibrin/fibrinogen degradation products; INR, international normalized ratio.

**Supplementary Table 3. Platelet counts, global markers of coagulation and fibrinolysis in patients with isolated traumatic brain injury with and without disseminated intravascular coagulation**

|  | 0h | 3h | 24h |
| --- | --- | --- | --- |
| Platelet counts (10^9^/L) |  |  |  |
| Non-DIC | 233(203-250) | 210(193-234) | 184(176-208) |
| DIC | 202(182-208) | 128(70-182)a | 140(101-190) |
| Prothrombin time (INR) |  |  |  |
| Non-DIC | 1.02(0.97-1.04) | 1.05(0.98-1.08) | 1.09(1.04-1.12) |
| DIC | 0.97(0.92-1.11) | 1.10(0.96-1.21) | 1.05(1.04-1.09) |
| APTT (sec) |  |  |  |
| Non-DIC | 26.1(24.8-27.5) | 27.2(25.1-30.6) | 29.5(27.6-31.6) |
| DIC | 24.2(23.3-28.0) | 29.7(25.0-31.7) | 30.6(27.7-32.7) |
| Fibrinogen (g/L) |  |  |  |
| Non-DIC | 2.47(2.29-2.79) | 2.28(202-275) | 3.22(283-388) |
| DIC | 2.31(1.63-2.89) | 1.86(1.16-2.44) | 3.39(2.21-3.95) |
| FDP (mg/L) |  |  |  |
| Non-DIC | 17.4(11.0-22.2) | 10.3(5.8-43.0) | 3.8(2.9-15.5) |
| DIC | 53.3(37.8-118.0)b | 43.7(25.7-106.4) | 30.8(6.7-32.7) |
| FDP/D-dimer ratio |  |  |  |
| Non-DIC | 2.29(1.57-3.12) | 2.03(1.47-2.51) | 1.93(1.77-2.51) |
| DIC | 2.38(1.70-3.16) | 2.03(1.60-4.09) | 2.57(2.21-2.65) |

APTT, activated partial thromboplastin time; DIC, disseminated intravascular coagulation; FDP, fibrin/fibrinogen degradation products; INR, international normalized ratio. a, *p*<0.05; b, *p*<0.01 vs. non-DIC.


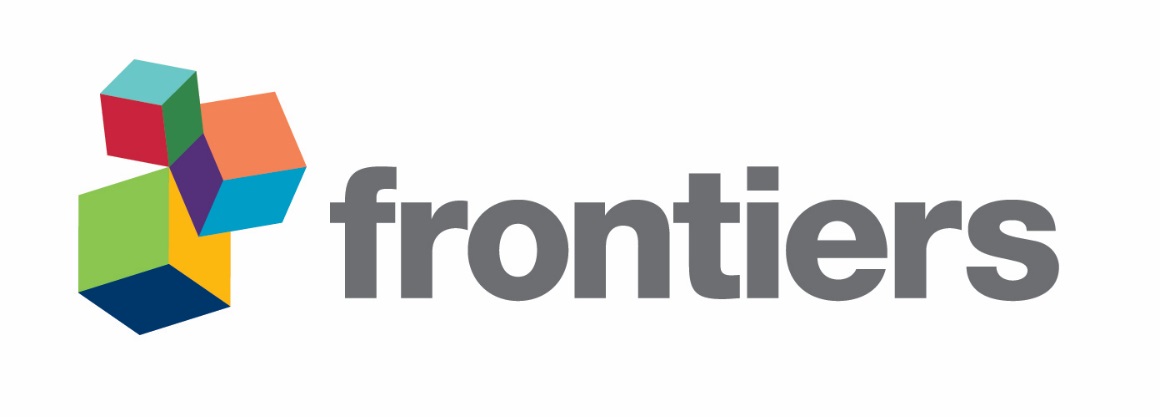

Supplement: Supplementary file 1 [file Data_Sheet_1.docx]
