# Supplementary figures and images for: Pathophysiology of Coagulopathy Induced by Traumatic Brain Injury Is Identical to That of Disseminated Intravascular Coagulation With Hyperfibrinolysis
Source: Front Med (Lausanne). 2021 Nov 15;8:767637. doi: 10.3389/fmed.2021.767637 (PMC8634586; doi:10.3389/fmed.2021.767637)

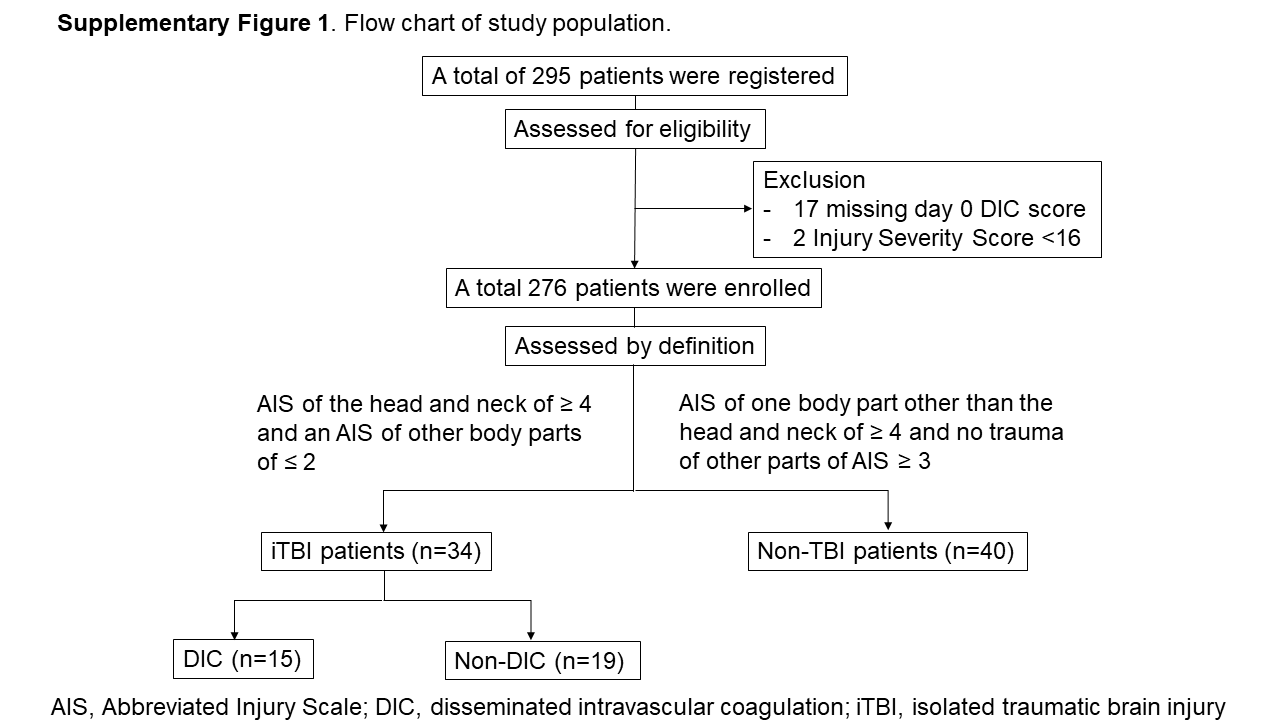

Supplement: Supplementary file 2 [file Image_1.TIF]
